# Cyanobacterial Algal Bloom Monitoring: Molecular Methods and Technologies for Freshwater Ecosystems

**DOI:** 10.3390/microorganisms11040851

**Published:** 2023-03-27

**Authors:** Faizan Saleem, Jennifer L. Jiang, Rachelle Atrache, Athanasios Paschos, Thomas A. Edge, Herb E. Schellhorn

**Affiliations:** Department of Biology, McMaster University, Hamilton, ON L8S 4L8, Canada

**Keywords:** cyanobacteria, harmful algal blooms, Great Lakes, cyanotoxins, microcystin, cyanobacteria lysis, molecular methods

## Abstract

Cyanobacteria (blue-green algae) can accumulate to form harmful algal blooms (HABs) on the surface of freshwater ecosystems under eutrophic conditions. Extensive HAB events can threaten local wildlife, public health, and the utilization of recreational waters. For the detection/quantification of cyanobacteria and cyanotoxins, both the United States Environmental Protection Agency (USEPA) and Health Canada increasingly indicate that molecular methods can be useful. However, each molecular detection method has specific advantages and limitations for monitoring HABs in recreational water ecosystems. Rapidly developing modern technologies, including satellite imaging, biosensors, and machine learning/artificial intelligence, can be integrated with standard/conventional methods to overcome the limitations associated with traditional cyanobacterial detection methodology. We examine advances in cyanobacterial cell lysis methodology and conventional/modern molecular detection methods, including imaging techniques, polymerase chain reaction (PCR)/DNA sequencing, enzyme-linked immunosorbent assays (ELISA), mass spectrometry, remote sensing, and machine learning/AI-based prediction models. This review focuses specifically on methodologies likely to be employed for recreational water ecosystems, especially in the Great Lakes region of North America.

## 1. Introduction

Cyanobacteria (blue-green algae) are a diverse group of bacteria that, in comparison to other bacterial communities, can uniquely perform photosynthesis and modulate the environmental oxygen content [1,2]. Prolific growth under eutrophic conditions leads to the accumulation of cyanobacterial biomass and the formation of algal blooms in freshwater ecosystems [3]. Freshwater algal blooms mainly comprise one or more of *Aphanizomenon, Cylindrospermopsis, Dolichospermum, Microcystis, Nodularia, Planktothrix,* and *Trichodesmium,* which have regulatory impacts on the ecological processes of aquatic ecosystems [3,4,5]. For example, cyanobacterial species that uniquely utilize carbon dioxide and nitrogen-dependent metabolism are essential nitrogen-fixing organisms under anaerobic conditions [6,7]. However, bloom-infested freshwater lakes may harbor cyanotoxin-producing cyanobacterial species, including *Microcystis, Dolichospermum* (formerly *Anabaena*)*, Raphidiopsis* (formerly *Cylindrospermopsis*)*,* and *Planktothrix*, which can impair water quality [8,9,10].

Favorable environmental factors, including high temperature and availability of micronutrients, allow the cyanobacterial blooms to propagate, leading to hypoxic/anoxic conditions as blooms decay and consume oxygen [11,12]. Aside from the over-population/propagation, cyanobacterial blooms can render freshwater ecosystems unsuitable for drinking water and recreational uses by producing metabolites and metabolic byproducts with unpleasant olfactory and gustatory properties [13,14,15,16]. The predominant density of cyano-toxigenic species in water bodies leads to the formation of harmful algal blooms (HABs). Microcystins, cylindrospermopsins, anatoxins, and saxitoxins are the most common cyanotoxins produced by harmful algal blooms, and doses as low as parts per billion can induce acute toxicity [17,18]. Toxigenic health effects of HAB cyanotoxins can manifest directly through ingestion or indirectly through consuming contaminated food products, including fish, mollusks, and agricultural produce [19,20]. Therefore, raw freshwater sources, including lakes and rivers, are monitored seasonally for their cyanotoxin potential to avoid health or economic losses. 

The North American Great Lakes, the largest freshwater system in the world, are important for drinking water, recreation, industry, and agriculture. Due to shallow depths and temperate weather, these lakes, especially Lake Erie, face seasonal large-scale harmful algal bloom events [21]. Although Lake Erie experiences the most extensive bloom events among the Great Lakes, cyanoHABs and cyanotoxins are now also present across all the Great Lakes [22,23,24,25]. Despite the geographical and economic significance of the Great Lakes, the dynamics of cyanobacterial bloom formation are poorly understood.

The water quality of the Great Lakes is monitored by both the United States Environmental Protection Agency (USEPA) and Environment and Climate Change Canada, as well as other Federal, State, and Provincial programs [26]. For cyanobacterial monitoring, USEPA and Health Canada identify commonly used methods based on microscopy, enzyme-linked immunosorbent assays (ELISA), protein phosphatase inhibition assays (PPIA), and high-performance liquid chromatography coupled with either mass spectrometry (LCMS) or ultraviolet/photodiode array detectors for cyanotoxin detection and quantification [27,28]. For microcystin detection in recreational waters, the USEPA recommends LCMS- and ELISA-based methods [29,30,31]. LCMS- and ELISA-based methods are highly sensitive to microcystin detection, but ELISA-based methods cannot distinguish microcystin congeners [29,30,31]. The USEPA and Health Canada also indicate molecular biology methodology, including conventional polymerase chain reaction (PCR), quantitative real-time PCR (qPCR), and microarrays/DNA chips that can be useful for cyanobacterial monitoring [27,28]. However, standardized molecular biology protocols have yet to be established for detecting harmful algal blooms or cyanotoxins. Alternative high-throughput techniques for assessing freshwater ecosystems, including atomic force microscopy (AFM) and biomonitoring, are currently available [32,33]. AFM can visualize microcystin strand formation in the presence of metal ions and can also examine the nanomechanical/adhesion properties of algal cells [34,35]. Biomonitoring can determine the nutrient and metal composition in water samples, which directly impacts algal populations [36]. This review focuses on the applications, advantages, and limitations associated with the molecular biological techniques for harmful algal bloom monitoring in recreational water ecosystems, including the Great Lakes. 

This review will evaluate new molecular methods for harmful algal bloom monitoring in the Great Lakes ecosystems. Objectives of this comparative review are: (1) assessing cell lysis methods for extracting biomolecules from bloom-infested water samples; (2) evaluating molecular methods for cyanobacterial and cyanotoxin detection; and (3) addressing the benefits and limitations associated with molecular methods.

## 2. Methods for the Disruption and Lysis of Algal Bloom Cyanobacteria

Cyanobacterial density can be monitored by direct (cell mass) or indirect (intracellular biomolecules) methods [37]. However, USEPA guidelines for monitoring cyanotoxin require data for the total cyanotoxin concentration, including both extracellular and intracellular concentrations [27]. Complete lysis of cyanobacterial cells is necessary to obtain an accurate estimate of intracellular cyanotoxin concentrations. Unlike the cell walls of other bacteria, cyanobacterial cell walls have a much thicker and highly crosslinked peptidoglycan layer, presenting a challenging obstacle for molecular detection methods that require cell lysis [38,39,40]. This section evaluates the mechanisms, limitations, and benefits of lysis methods for cyanobacterial cells.

### 2.1. Chemical Cell Lysis

Chemical lysis employs detergents, enzymes, or organic solvents to disrupt the cell membrane. Detergents are the most common chemical agents that break down non-covalent interactions for cell lysis [41]. However, using a detergent-only lysis buffer may be insufficient; for *Microcystis aeruginosa*, this method only disrupts 37% of the total cell wall material (Table 1) [42]. For cyanobacterial cells, the addition of proteolytic enzymes (proteinase K and lysozyme) and reducing agents (dithiothreitol and β-mercaptoethanol) can further disrupt stabilizing covalent interactions and increase the lysis efficiency to near 100% (Table 1) [43]. Chemical lysis methods yield a high recovery of intact nucleic acids with lower contamination from non-target biomolecules [43,44,45]. Evaluation of DNA extraction following chemical lysis with a chemical lysis buffer (Tris-Urea) provided yields from 230 to 479 µg/mL of intact DNA from species of *Rivularia*, *Dolichospermum, Synechocystis*, and *Synechococcus* [46]. 

Chemical lysis can provide a higher yield of the targeted biomolecule. However, it can also result in chemical contamination that can cause interference in the downstream analysis [47,48]. Detergents (e.g., Triton-X 100) or organic solvents (e.g., phenol) degrade or stop the DNA polymerase from binding to the DNA template during PCR [49]. Hence, the greater yield of DNA from enzyme-based methods may be negated by the potential inhibition of downstream diagnostic protocols, including PCR [49]. Additionally, contaminating phenol absorbs at 230 nm UV, which may lead to over-estimation or false positives [50].

### 2.2. Ultrasonic Cell Lysis

Ultrasonication uses rapidly changing sonic pressure to cause cavitation, which agitates and disrupts cellular membranes and cell walls [51,52]. For some filamentous strains, sonication can break down the filaments into smaller structures or single cells [53,54]. In the treatment of smaller samples, sonication can be achieved through an ultrasonic bath or probe, with the latter being more efficient in lysis [55] (bath sonication of *Microcystis aeruginosa* resulted in 73% lysis after 30 min (Table 1) [42], while probe sonication can yield 80% lysis after 5 min (Table 1) [56]).

An advantage of ultrasonic lysis is the elimination of potential chemical contaminants (associated with chemical lysis), so there is little interference of downstream diagnostic PCR assays by enzymatic inhibition. Although probe sonication increases the cell lysis efficiency with reduced sonication time, a fraction of cells can remain intact even with an increase in sonication time, and overexposure to sonication can cause nucleic acid fragmentation [46,56]. In addition, cellular debris and biomolecules remain in the lysate, which can interfere with subsequent analytical techniques [49]. For cyanobacterial cells, a longer processing time and subsequent purification steps may be required to obtain higher yield and purity [55]. 

**Table 1:** Cyanobacterial cell lysis methods.

Lysis Method		Lysis Efficiency	Advantages	Limitations
Chemical	Detergent	37% [43]	High efficiency, high yield, low DNA degradation	Deposit contaminants that interfere with downstream assays
	Detergent-enzyme cocktail	100% [43]		
Ultrasonic	Bath sonication	73% [42]	Avoids chemical contaminants, increases purity of extracted biomolecule	Long processing time, incomplete lysis, DNA shearing
	Probe sonication	80% [56]		
Mechanical	Bead beating	50–99% [42,43]	Avoids chemical contaminants, increases purity of extracted biomolecule	DNA shearing (requires optimal bead beating parameters), inconsistent lysis efficiency depending on cell morphology
Cryogenic	Freeze-thaw	19–100% [42,56]	Avoids chemical contaminants, increases purity of extracted biomolecule	Inconsistent lysis efficiency
	Lyophilization	92–98% [42]	High efficiency, avoids chemical contaminants, increases purity of extracted biomolecule	Long waiting times can limit the use of rapid detection methods

### 2.3. Physical Cell Lysis

Physical cell lysis methods apply external forces, including bead beating, to rupture the cell membrane and cell wall. The types of physical lysis discussed can be divided into two categories: mechanical and cryogenic (Table 1). A commonly used method of mechanical lysis is bead beating, for which ceramic or glass beads are agitated together with the cell sample to achieve cell lysis [57]. Bead beating for 3 min with 0.5 mm glass beads can result in the lysis of 99% of *M. aeruginosa* cells (Table 1) [42]. However, for *Synechocystis*, only 50% of the total cells can be lysed after bead beating [43]. Cryogenic methods include freeze–thawing and lyophilization. During cycles of freezing in either dry ice or a freezer, cell swelling and ice formation on the cellular membrane and cell wall cause structural disruption when the samples are thawed [41]. Lyophilization is similar in principle in that cyanobacterial samples are frozen and then dehydrated using a freeze-dryer under a vacuum [42]. Lysis is ultimately achieved upon rehydration [42]. For *M. aeruginosa*, three freeze–thaw cycles (−70 °C for 10 min, then 37 °C for 5 min) resulted in the lysis of only 19% of total cells (Table 1) [42]. However, another trial demonstrated that almost no *M. aeruginosa* cells remained intact after one extensive freeze–thaw cycle (−20 °C for 12 h, then 25 °C for 2–4 h) (Table 1) [56]. On the other hand, lyophilization had very high efficiency, disrupting 92% and 98% of total cells after rehydration with deionized water and methanol, respectively (Table 1) [42].

Similar to ultrasonication, physical cell lysis methods also circumvent the issue of chemical contamination. Despite the inconsistencies in efficiency, bead beating remains a standard method for most cell lysis protocols [58,59,60,61]. DNA extraction from bead-beaten benthic cyanobacteria resulted in a concentration of 15 µg/mL [55], a lower recovery than the chemical lysis method. The efficiency of bead beating can differ widely depending on the morphology and characteristics of the cyanobacterial cells (e.g., benthic species of cyanobacteria are challenging to disrupt due to their protective sheaths and mucilage [55]). For bead beating, the yield of the biomolecules can be increased by adjusting parameters, including beat size, bead count, and time [62,63]. Non-optimized parameters can lead to extensive DNA shearing, which may compromise the integrity of downstream analysis [46]. Compared to bead beating, cryogenic lysis, particularly lyophilization, provides high lysis efficiency approximately equal to that of chemical lysis with an optimized buffer cocktail [42,46]. Despite the higher lysis efficiency, lyophilization requires a long waiting time (upwards of days) for the freeze drying to be complete [42], thus limiting the application for routine, rapid monitoring.

### 2.4. Combinatorial Cell Lysis Methodologies

Lysis efficiency depends on the characteristics and morphology of the cells [64]. For cyanobacteria, employing several lysis methods in succession may be helpful to ensure maximum lysis or to break down cells of highly resilient species. Combining bead beating with an optimized chemical lysis cocktail can achieve a 2–3-fold increase in efficiency for *Synechocystis* and *Synechococcus* spp., allowing for maximal lysis of cyanobacterial cells [43]. In addition, combining cryogenic and enzyme lysis methods increases DNA yield for filamentous *Arthrospira* species [65]. Additionally, including chemical lysis buffer, lysozyme, and proteinase K to bead beating can provide a 2-fold increase in extracted DNA compared to bead beating alone [55]. Complementarity of cell lysis methods with each other can increase lysis efficiency and DNA yield, as well as reduce the limitations associated with each method.

## 3. Methods and Technologies for Cyanobacterial and Cyanotoxin Monitoring

Conventional strategies include biological assays and chromatographic technologies for determining cyanotoxin concentration, as well as quantitative enumeration of cyanobacterial cells. Recent advancements in molecular biology and computer science led to rapid improvements in PCR, DNA sequencing, microfluidics, and machine learning methods to probe and monitor cyanobacterial density/activity in recreational waters. This section will introduce methods for cyanobacteria/cyanotoxin monitoring, evaluate their current advantages and disadvantages, and evaluate each method’s sensitivity or specificity in detecting harmful algal blooms. The focus is on the diagnostic molecules of microcystins and DNA, as they remain stable after extraction [66]. The sensitivities, benefits, and limitations of cyanotoxin quantification methods are summarized in Table 2, while those of cyanobacterial cell quantification methods are summarized in Table 3.

### 3.1. Conventional Methods and Techniques

The simplest protocol for cyanobacterial cell counting by microscopy is the Utermöhl method (for phytoplankton), in which sedimentation of cyanobacterial cells is performed before enumeration using an inverted microscope [67,68]. In addition to simplicity, microscopic methods can provide high specificity and allow for identification up to the genus and species levels [69,70]. However, microcystin levels are only weakly correlated with microscopic cell counts and may not fully account for the cyano-toxicity of the recreational waters [71]. Direct enumeration can also be time-consuming [70]; the Utermöhl method requires 24–48 h of sedimentation time, limiting its applicability to real-time, rapid monitoring [67,68]. Enumeration and identification of cyanobacteria via microscopy also require trained expertise in the field, and reliability can significantly vary depending on the analyst’s skill. Expertise limitation for microscopic cyanobacterial counting can be overcome by integrating computational models (e.g., PhytoNumb3rs [72]) or automated microscopy (e.g., FlowCam Cyano (Yokogawa Fluid Imaging Technologies, Scarborough, ME, USA)) for cyanobacterial morphology. Atomic force microscopy can visualize even a single molecule in ultra-high resolution and can be used along with conventional microscopic techniques to robustly examine the properties of both microcystin and cyanobacterial cells [32,34,35]. AFM enables assessments in liquid media that mimic intracellular environments. However, AFM requires sample immobilization [73,74]. Overall, quantitative enumeration can provide a suitable reference for more advanced monitoring technologies, but it can be somewhat inconsistent and timely when used by itself.

Enzyme-linked immunosorbent assays (ELISAs) can be used to quantify cyanotoxins using competitive binding between antibodies and the targeted toxins [75]. The current standard for microcystin detection is an assay that recognizes the β-amino acid, ADDA, (4E, 6E 3-amino-9-methoxy-2, 6, 8-trimethyl-10-phenyldeca-4, 6-dienoic acid) that is present in most of the penta- and heptapeptide congeners of microcystins and nodularins [76,77]. The ADDA-based ELISA method is rapid (2–3 h) and can be performed without pre-concentration of lake water samples. It is highly sensitive, being able to detect a minimum of 0.02–0.07 ng/mL of microcystins (Table 2), which is below the Health Canada guideline for microcystin-LR of 1.5 ng/mL [78]. Commercially available ADDA-microcystin ELISA kits (Manuals available at: https://www.enzolifesciences.com/ALX-850-319/microcystins-adda-specific-elisa-kit/ (accessed on 18 February 2023) and https://www.caymanchem.com/product/502000/microcystin-elisa-kit (accessed on 18 February 2023)) report sensitivities ranging from 0.1–0.196 ng/mL (Enzo Life Sciences, Inc., New York, NY, USA, Cayman Chemical, Ann Arbor, MI, USA) (Table 2). USEPA testing of the Abraxis ELISA Microcystins Strip Test (Gold Standard Diagnostics Horsham Inc. Warminster, PA, USA) provided a minimum reporting limit of 0.30 ng/mL (Table 2) [31]. Commercially available kits can vary in terms of detection sensitivity/limit. Therefore, a comparative validation of ELISA kits should be performed to determine the optimal sensitivity for a recreational water ecosystem. One limitation of ELISA-based methods is their inability to distinguish between different congeners of microcystins and nodularins present in a sample [76,79]. Lack of specificity is reflected in common cross-reactions with microcystin degradation byproducts and metabolites, which can lead to overestimation/false-positive of cyano-toxicity [80,81,82,83]. 

Chromatography purifies compounds (including cyanotoxins) based on hydrophobic, hydrophilic, ionic, or affinity-based mobility through a separation medium [84]. It can be coupled with a spectrophotometric detection method for quantification [18]. Liquid chromatography–mass spectrometry (e.g., liquid chromatography–electrospray ionization–high-resolution mass spectrometry, ultra-high-performance liquid chromatography–high-resolution mass spectrometry) can provide a sensitivity as low as 0.000004–0.02 ng/mL (Table 2) [85,86]. Aside from lower detection limits (high sensitivity), chromatography coupled with tandem mass spectrometry can differentiate between different cyano-toxin congeners [87]. As such, chromatography-coupled–mass spectrometry methods are highly sensitive and can resolve specific congeners of interest. Although, it is essential to note that chromatographic methods require specific congener standards for qualitative/quantitative assessment (pure quantity of targeted molecule). However, standards for only a few naturally occurring microcystins are commercially available [80,88,89]. Additionally, chromatography requires highly trained personnel and high recurrent costs [90]. Overall, most conventional methods have limitations that can be overcome by integrating or coupling modern molecular biological or computational methods. 

### 3.2. Molecular Methods and Techniques

#### 3.2.1. Polymerase Chain Reaction (PCR) and DNA Sequencing

Conventional polymerase chain reaction (PCR) uses amplification of organism-specific, genomic DNA sequences for qualitative (absence or presence) analysis, while real-time PCR or quantitative PCR incorporates DNA binding dye or probe to quantify the DNA target (cyanobacterial or cyanotoxin gene copy number) in purified DNA (e.g., DNA extracted from an environmental sample, including algal blooms) [91]. Newer digital PCR techniques that partition purified DNA samples into thousands of oil droplets or wells on a chip to quantify the DNA target are also available. The expression of actively transcribed cyanotoxin genes can be assessed using reverse transcription qPCR (RT-qPCR) to distinguish active from quiescent cells [92,93]. Universal gene target (16s rRNA) analysis, which has a 97% conserved region for cyanobacteria, is commonly used for qPCR studies [94,95,96,97]. One assessment reported a detection limit of twenty-five gene copies per reaction (Table 3) [98]. Although the 16s rRNA gene effectively quantifies cyano-toxigenic cyanobacteria, including *Microcystis* and *Planktothrix* spp. [77], it is not specific enough for all cyanobacteria and can result in overestimation or false positives [99,100]. Attempts have been made to counter non-specificity by using cyanobacterial barcoding (integrating PCR amplification and next-generation DNA sequencing ), which can allow the use of downstream bioinformatics to obtain a higher confidence level of cyanobacterial identification [101,102,103]. 

For cyanotoxin detection/quantification, the *mcyE* (microcystin gene cluster) assay detects all potential microcystin producers and the nodularin synthetase gene clusters [104]. The *mcyA* assay covers many, but not all, microcystin-producing strains [9], while the *mcyE* and *mcyG* assays are sensitive and specific to *Microcystis* and *Planktothrix* [105,106,107]. The detection limit for the *mcyE* assay was reported to be 3–63 gene copies per PCR reaction (Table 2) [108]. To increase the PCR detection specificity, conserved signature proteins (CSPs) and conserved signature indels (CSIs) that are highly specific to a particular clade of organisms can be used [109]. Thirty-nine cyanobacteria-specific conserved signatures proteins have been identified for cyanobacteria species [110]. These can be used for generating cyano-specific PCR/qPCR methods.

For application in the Great Lakes ecosystems, the USEPA and Health Canada have identified PCR and qPCR as useful methods for detecting cyanobacteria and cyanotoxins [27,28]. PCR methodology has been used for qualitative analysis of bloom-infested Lake Erie to determine that up to 42% of the total cyanobacterial population comprises *Microcystis*, and the measured proportion of total *Microcystis* demonstrates a strong correlation with total microcystin concentrations [98,111]. qPCR and RT-qPCR have recently been demonstrated to be useful early warning tools for cyanobacterial blooms and cyanotoxin production in inland lakes in Ohio [77,112]. Characterization of the spatiotemporal variation associated with cyanobacterial blooms in Lake Erie has also been assessed using PCR, followed by high-throughput sequencing of the 16s rRNA gene [113]. These applications indicate the potential of using PCR/qPCR and DNA sequencing for cyanobacterial identification in the Great Lakes ecosystems.

Compared to traditional methods, including microscopic enumeration, qPCR can be more rapid and provide results within 3–4 h [77,90]. In addition, by incorporating the cyanotoxin gene quantification, the cyanotoxin potential of recreational waters, including the Great Lakes, can be determined [77]. A limitation of PCR is the potential to be inhibited by environmental contaminants, including cell debris, humic acids, detergents, and polysaccharides. These contaminants can degrade or sequester the DNA polymerase and nucleic acids, thus inhibiting PCR amplification [49]. Furthermore, DNA extraction efficiency can also be a limiting factor in PCR analysis. The efficiency of DNA recovery in microbial identification can be approximately 30% or less due to the environmental matrix interferences and unique resilience of the cyanobacterial cell wall and sheath [91]. Additionally, when using specific target genes for PCR, unknown toxigenic species with environmental variations in the gene sequence may go undetected (under-representation of the counts) [114,115]. On the other hand, non-viable cells can get detected when using universal targets (over-representation of the data) [90]. While these are challenges for PCR approaches, they can also be addressed through rigorous quality control steps, as demonstrated by the development of USEPA’s standard *Enterococcus* qPCR method now being applied at Great Lakes beaches [116,117]. In conclusion, PCR-based techniques can efficiently and conveniently monitor cyanobacterial quantity and toxicity, and the associated limitations can be resolved by integrating other molecular methods.

#### 3.2.2. Microfluidic and DNA Capture Devices

Microfluidic and DNA capture devices incorporate target-specific probes or fluorophores that can bind to the target molecule in the cellular/DNA extract and generate a signal corresponding to the amount of the targeted molecule [118,119]. A handheld microfluidic device capable of rapid and onsite detection and identification of microcystins and other cyanotoxins is available [120,121]. The device performs an automated ELISA assay, as a disposable microfluidic cartridge, with a detection limit of 0.16 ng/mL (Table 2), comparable to commercial ELISA kits for laboratory settings [120]. An antibody microarray chip called CYANOCHIP Is available for cyanobacterial detection in soil and water samples. It has a detection limit of 100–1000 cells (Table 3) [122,123].

Based on DNA sequence detection, a microfluidic chip biosensor targeting the cyanopeptolin (a cyanotoxin produced by *Planktothrix* and *Microcystis* spp.) can provide a detection limit of 6 × 10^−12^ M of target DNA [124]. However, testing was only conducted with purified PCR products for this sensor. Similarly, a cantilever biosensor assay designed to recognize a conserved region of the 16s rRNA gene in *M. aeruginosa* can detect approximately 50 cells/mL in purified DNA and 500 cells/mL for river water samples (Table 3) [125]. DNA chip assays are also available for both the 16s rRNA and *mcyE* genes to monitor microcystin levels [126,127]. These have been tested for environmental samples, and the detection limit is as low as 1–5 fmol of DNA (Table 2 and Table 3) [126].

A significant advantage of microf”Iid’c and chip assays is their portability and minimum sample volume [128]. The devices are also capable of providing rapid detection of the target molecules. For example, the CYANOchip takes 3 h, including incubation time, to provide results [123]. For the microarray chip, multiple targets can be monitored and analyzed simultaneously using 17 antibodies [123], further enhancing its efficiency. These factors all increase the potential for onsite microfluidics and chip technology applications. However, testing on environmentally complex samples can reduce the sensitivity due to the presence of background microorganisms [125]. A possible extension to microfluidic devices is the biomonitoring tool, which determines analytes impacting algal growth with high sensitivity and low sample volumes [36]. However, it is often difficult to directly apply the results of biomonitoring to make predictions on freshwater ecosystems [33,36]. Overall, microfluidic and DNA capture devices can provide portability, but their usage should be incorporated with the other molecular methods to obtain higher sensitivity. 

**Table 2:** Cyanotoxin (microcystin) detection and quantification methods.

Method	Sensitivity	Advantages	Limitations
Enzyme-linked immunosorbent assay (ELISA)	0.02–0.30 ng/mL [31,76]	Rapid, high sensitivity, the limit of detection within Health Canada guidelines	Low specificity, congener-independent, cross-reaction with cyanotoxin metabolites lead to false positives/overestimation
Liquid chromatography-mass spectrometry (LC/MS)	0.000004–0.02 ng/mL [85,86]	High sensitivity, the limit of detection within Health Canada guidelines, congener-specific	Few standards are commercially available, require highly trained personnel, high recurrent cost
Quantitative polymerase chain reaction (qPCR) targeting *mcyE*	3–63 gene copies per reaction [108]	Rapid, allows both qualitative and quantitative analysis, allows assessment of cyanotoxin potential	Environmental contaminants can inhibit amplification, species with sequence variations may go undetected, detection of non-viable cells
Microfluidic device	0.16 ng/mL [120,121]	Rapid, portable, and suitable for the field use, requires minimal sample volume	Reduced sensitivity, interference from sample background
DNA capture targeting *mcyE*	1–5 fmol of DNA [126]	Rapid, convenient, potential for field use, requires minimal sample volume	Reduced sensitivity, interference from sample background, limited testing on complex environmental samples.

## 4. Future Directions

### 4.1. Remote Sensing/Satellite Imaging

Satellite imaging and remote sensing algorithms allow coverage of a larger geographical area than the methods based on water sample collection. Standard sensors for monitoring bloom dynamics include Landsat, Moderate Resolution Imaging Spectroradiometer (MODIS), Medium Resolution Imaging Spectrometer (MERIS), and Ocean and Land Color Instrument (OLCI) due to their optimum temporal, spatial, and spectral resolutions [70,129]. A novel method is available to determine the magnitude of cyanoHABs across the United States using data from MERIS; the method has been extended to OLCI [130]. The current Cyanobacteria Index (CI), which uses MERIS and OLCI data, identifies cyanotoxin-producing cyanobacteria with 84% accuracy and a detection limit of 1 CI value (determined based on chlorophyll-a absorption at and around 681 nm) (Table 3) [131,132]. Cyanotoxins cannot be directly measured using remote sensing, as the concentration can only be determined indirectly by detecting the surrogate pigments, including phycocyanin or chlorophyll-a [133]. To appropriately measure these pigments, band ratio algorithms in the red and near-infrared spectral regions can be employed [129]. Evaluation of twenty-seven algorithms for cyanobacterial biovolume determination indicates that both chlorophyll-a- and phycocyanin-based algorithms achieved high detection accuracy, with phycocyanin being slightly more sensitive than chlorophyll methods [134].

Remote sensing can especially be useful for inland water bodies [129], including the Great Lakes. The Cyanobacteria Assessment Network (CyAN) project by the USEPA aims to develop an early warning indicator system for cyanobacterial blooms in freshwater systems using satellite data records [135]. The project utilizes products for MERIS, OLCI, as well as MODIS. For this purpose, the potential for phytoplankton exposure at a Great Lakes beach was determined using two remote sensing methods: the MODIS ocean chlorophyll-a algorithm and the MERIS CI. As part of the EOLakeWatch (Earth Observation Lake Watch) program, Environment and Climate Change Canada monitors the water quality of lakes across Canada (including the Great Lakes) using satellite imagery [136]. Chlorophyll-a algorithms are applied to MERIS and OLCI data, from which algal bloom indices are derived [137].

There are specific challenges accompanying the application of remote sensing for bloom monitoring, a major one being the need to identify surrogate pigments. Variability in the correlation between chlorophyll-a/phycocyanin and cyanotoxins can make it difficult to accurately infer water toxicity from satellite imagery, especially for microcystins [133]. Remote sensing is also limited to water systems that cover large surface areas, and it may be inaccessible for smaller, more secluded areas for recreation [70]. Additionally, differing cyanobacteria composition and optical properties in parts of Lake Erie can lead to misidentification by remote sensing algorithms [138]. Although remote sensing and satellite imaging provide a broader geographical range of detection, this methodology may be unable to distinguish between harmful algal blooms and other cyanobacterial biomass. Integrating remote sensing with cyanotoxin quantification can overcome the specificity limitation and provide more robust information. 

### 4.2. Artificial Intelligence and Machine-Learning-Based Prediction Tools

Following the ever-increasing volume of available data on aquatic ecosystems, artificial intelligence and machine-learning algorithms can become valuable tools in making predictions on cyanobacterial bloom activities. There are multiple ways in which these algorithms can be applied to cyanobacterial monitoring, including cell imaging and water quality prediction deep learning algorithms [139,140,141,142]. Microscopic images of cyanobacterial samples can be processed for enumeration and species identification using imaging-based detection software. A fast regional convolutional neural network (R-CNN) was able to identify five major species of cyanobacteria (including *Microcystis* spp.), with average precision values ranging from 0.890 to 0.973 [141]. In the same study, a basic convolutional neural network (CNN) was able to quantify populations of 50–250 cyanobacterial cells accurately (Table 3). Another deep learning-based method for qualitative microscopic image processing using a convolution fusion network (CFN) outperformed classic CNN models in terms of accuracy with a prediction rate of 99.36% in classifying cyanobacterial cells [142]. A multi-objective hybrid evolutionary algorithm (MOHEA) can provide crucial threshold exceedances of local cyanobacterial outbreaks and forecast cyanobacterial activity seven days before bloom events [139]. Chlorophyll-a, a compound necessary for photosynthesis, can be used to train machine-learning algorithms and predict the onset of cyanobacterial blooms. For example, an auto-regressive integrated moving average (ARIMA) model was developed to predict the chlorophyll-a level in Lake Taihu, China. This model demonstrates the potential for use as a cyanobacterial bloom warning system [140].

Unlike conventional or molecular biology methods, models rooted in artificial intelligence and machine learning can solve more complex problems with a greater number of variables [143]. Once proper algorithms have been developed and implemented, it can reduce the need for onsite expertise in cyanobacterial monitoring [70]. However, these technologies also possess many challenges in their current state of development. For example, to reach an accurate and reliable prediction, it is necessary to integrate multiple algorithms [144]. The dataset size needed is also an issue, as most collected data (70–80%) are used as a training set [145]. Only the remaining 20–30% is used as test data to measure prediction accuracy, creating a need for an immensely more extensive and diverse dataset to observe all the possible environmental patterns [145]. The AI/machine learning models produced under such strict guidelines may not be applicable across geospatial differences or in environments with drastically different physical and chemical differences. These limitations must first be addressed for the widespread adoption of AI and machine learning technologies to be feasible.

**Table 3:** Cyanobacterial detection and quantification methods.

Method	Sensitivity	Advantages	Limitations
Microscopic enumeration	Not applicable	Simplicity, identification up to the genus and species levels	Time-consuming, requires trained personnel, and accuracy is dependent on the skill of the analyst
Quantitative polymerase chain reaction (qPCR) targeting 16 s rRNA	25 gene copies per reaction [98]	Rapid, allows both qualitative and quantitative analysis	Non-specificity of a target gene, environmental contaminants inhibit amplification, detection of unviable cells
Antibody microarray chip (CYANOCHIP)	100 cells [122,123]	Rapid, convenient, potential for field use, requires minimal sample volume	Reduced sensitivity, interference from sample background
Biosensor assay	50–500 cells/mL [125]	Rapid, convenient, potential for field use, requires minimal sample volume	Reduced sensitivity, interference from sample background
DNA capture device targeting 16 s rRNA	1–5 fmol of DNA [126]	Rapid, convenient, potential for field use, requires minimal sample volume	Reduced sensitivity, interference from sample background
Remote sensing	1 Cyanobacteria Index (CI) value [132]	Extensive coverage of the geographical area, useful for inland bodies of water	Variability in the correlation between surrogate pigment and toxicity, inaccessible for smaller areas, and differing cyanobacterial composition leads to misinterpretation
Artificial intelligence (convolutional neural network)	50 cells [141]	Capable of complex analysis, reducing the need for onsite expertise	Need to integrate multiple algorithms for high reliability, requires extensive and diverse datasets, not applicable across geospatial differences

## 5. Conclusions

Cyanobacterial harmful algal blooms threaten the conservation of essential freshwater ecosystems, including the North American Great Lakes. Conventional/standard molecular methods for cyanobacterial detection are available. However, specific limitations are associated with each method.

1. Cyanobacterial cells resist disruption methods making quantitative recovery of potential diagnostic molecules difficult. However, optimizing protocol parameters and combining multiple lysis methods can lead to complete disruption
2. ELISA-based toxin detection methods (including microfluidic devices) alone cannot resolve or quantify all microcystin congeners. Chromatography–mass spectrometry methods can unambiguously identify microcystins and other cyanotoxins. However, the obtained information is difficult to incorporate into a public health response due to the lack of commercially available standards.
3. DNA diagnostic methods (PCR, DNA capture devices) targeting the 16 s rRNA gene, while useful for other bacteria, is of limited value for cyanobacteria due to insufficient specificity. This obstacle can be countered by metabarcoding or targeting a toxin gene such as the *Mcy* (microcystin synthetase) gene cluster.
4. Bloom monitoring may be aided by novel technologies, particularly in quickly establishing spatiotemporal characteristics of specific events. These technologies can augment traditional characterization methods in producing a public health response. However, while ongoing, standardization of common tools for this ancillary information still needs to be completed.
5. Newer/modern technologies, including satellite imaging, biosensors, and machine learning/artificial intelligence methods, can be integrated with the conventional/standard molecular methods to overcome the problems associated with cyanobacterial detection in recreational water ecosystems, including the Great Lakes.

## Data Availability

Data sharing not applicable.

## References

[1] Bekker A., Holland H.D., Wang P.-L., Rumble D., Stein H.J., Hannah J.L., Coetzee L.L., Beukes N.J. (2004). Dating the Rise of Atmospheric Oxygen. Nature.

[2] Sánchez-Baracaldo P., Cardona T. (2020). On the Origin of Oxygenic Photosynthesis and Cyanobacteria. New Phytol..

[3] Rastogi R.P., Madamwar D., Incharoensakdi A. (2015). Bloom Dynamics of Cyanobacteria and Their Toxins: Environmental Health Impacts and Mitigation Strategies. Front. Microbiol..

[4] Huisman J., Codd G.A., Paerl H.W., Ibelings B.W., Verspagen J.M.H., Visser P.M. (2018). Cyanobacterial Blooms. Nat. Rev. Microbiol..

[5] Hoiczyk E., Hansel A. (2000). Cyanobacterial Cell Walls: News from an Unusual Prokaryotic Envelope. J. Bacteriol..

[6] Bothe H., Schmitz O., Yates M.G., Newton W.E. (2010). Nitrogen Fixation and Hydrogen Metabolism in Cyanobacteria. Microbiol. Mol. Biol. Rev. MMBR.

[7] Paerl H. (2017). The Cyanobacterial Nitrogen Fixation Paradox in Natural Waters. F1000Research.

[8] Van Dolah F.M. (2000). Marine Algal Toxins: Origins, Health Effects, and Their Increased Occurrence. Environ. Health Perspect..

[9] Hisbergues M., Christiansen G., Rouhiainen L., Sivonen K., Börner T. (2003). PCR-Based Identification of Microcystin-Producing Genotypes of Different Cyanobacterial Genera. Arch. Microbiol..

[10] Plaas H.E., Paerl H.W. (2021). Toxic Cyanobacteria: A Growing Threat to Water and Air Quality. Environ. Sci. Technol..

[11] Rabalais N.N., Díaz R.J., Levin L.A., Turner R.E., Gilbert D., Zhang J. (2010). Dynamics and Distribution of Natural and Human-Caused Hypoxia. Biogeosciences.

[12] Paerl H.W. (2017). Controlling Harmful Cyanobacterial Blooms in a Climatically More Extreme World: Management Options and Research Needs. J. Plankton Res..

[13] Izaguirre G., Taylor W.D. (2004). A Guide to Geosmin- and MIB-Producing Cyanobacteria in the United States. Water Sci. Technol. J. Int. Assoc. Water Pollut. Res..

[14] Jüttner F., Watson S.B. (2007). Biochemical and Ecological Control of Geosmin and 2-Methylisoborneol in Source Waters. Appl. Environ. Microbiol..

[15] Wang Z., Song G., Li Y., Yu G., Hou X., Gan Z., Li R. (2019). The Diversity, Origin, and Evolutionary Analysis of Geosmin Synthase Gene in Cyanobacteria. Sci. Total Environ..

[16] Watson S.B., Ridal J., Boyer G.L. (2008). Taste and Odour and Cyanobacterial Toxins: Impairment, Prediction, and Management in the Great Lakes. Can. J. Fish. Aquat. Sci..

[17] Miller T.R., Beversdorf L.J., Weirich C.A., Bartlett S.L. (2017). Cyanobacterial Toxins of the Laurentian Great Lakes, Their Toxicological Effects, and Numerical Limits in Drinking Water. Mar. Drugs.

[18] Svirčev Z., Lalić D., Savić G.B., Tokodi N., Backović D.D., Chen L., Meriluoto J., Codd G.A. (2019). Global Geographical and Historical Overview of Cyanotoxin Distribution and Cyanobacterial Poisonings. Arch. Toxicol..

[19] Ferrão-Filho A.d.S., Kozlowsky-Suzuki B. (2011). Cyanotoxins: Bioaccumulation and Effects on Aquatic Animals. Mar. Drugs.

[20] Shi L., Du X., Liu H., Chen X., Ma Y., Wang R., Tian Z., Zhang S., Guo H., Zhang H. (2021). Update on the Adverse Effects of Microcystins on the Liver. Environ. Res..

[21] Steffen M.M., Belisle B.S., Watson S.B., Boyer G.L., Wilhelm S.W. (2014). Status, Causes and Controls of Cyanobacterial Blooms in Lake Erie. J. Gt. Lakes Res..

[22] Makarewicz J.C., Boyer G.L., Lewis T.W., Guenther W., Atkinson J., Arnold M. (2009). Spatial and Temporal Distribution of the Cyanotoxin Microcystin-LR in the Lake Ontario Ecosystem: Coastal Embayments, Rivers, Nearshore and Offshore, and Upland Lakes. J. Gt. Lakes Res..

[23] Almuhtaram H., Cui Y., Zamyadi A., Hofmann R. (2018). Cyanotoxins and Cyanobacteria Cell Accumulations in Drinking Water Treatment Plants with a Low Risk of Bloom Formation at the Source. Toxins.

[24] Bartlett S.L., Brunner S.L., Klump J.V., Houghton E.M., Miller T.R. (2018). Spatial Analysis of Toxic or Otherwise Bioactive Cyanobacterial Peptides in Green Bay, Lake Michigan. J. Gt. Lakes Res..

[25] Sterner R.W., Reinl K.L., Lafrancois B.M., Brovold S., Miller T.R. (2020). A First Assessment of Cyanobacterial Blooms in Oligotrophic Lake Superior. Limnol. Oceanogr..

[26] United States Environmental Protection Agency; Environment and Climate Change Canada 2022 Progress Report of the Parties. Pursuant to the 2012 Canada-United States Great Lakes Water Quality Agreement. Cat. No.: En161-25E-PDF. EPA 905R22003. https://binational.net/wp-content/uploads/2022/07/2022-Progress-Report-of-the-Parties.pdf.

[27] United States Environmental Protection Agency Determination of Cyanotoxins in Drinking and Ambient Freshwaters. https://www.epa.gov/cyanohabs/determination-cyanotoxins-drinking-and-ambient-freshwaters.

[28] Health Canada Guidelines for Canadian Recreational Water Quality—Cyanobacteria and Their Toxins. https://www.canada.ca/en/health-canada/services/publications/healthy-living/guidance-canadian-recreational-water-quality-cyanobacteria-toxins.html.

[29] Shoemaker J.A., Tettenhorst D.R., de la Cruz A. Method 544. Determination of Microcystins and Nodularins in Drinking Water by Solid Phase Extraction and Liquid Chromatography/Tandem Mass Spectrometry (LC/MS/MS) 2015. https://cfpub.epa.gov/si/si_public_record_report.cfm?Lab=NERL&dirEntryId=306953&simpleSearch=1&searchAll=544.

[30] Shoemaker J.A., Tettenhorst D.R., de la Cruz A. Single Laboratory Validated Method for Determination of Microcystins and Nodularin in Ambient Freshwaters by Solid Phase Extraction and Liquid Chromatography/ Tandem Mass Spectrometry (LC/MS/MS) 2017. https://www.epa.gov/sites/default/files/2017-11/documents/microcystin_method_for_ambient_water_nov_2017.pdf.

[31] Zaffiro A., Rosenblum L., Wendelken S.C. Method 546: Determination of Total Microcystins and Nodularins in Drinking Water and Ambient Water by Adda Enzyme-Linked Immunosorbent Assay 2016. https://www.epa.gov/sites/default/files/2016-09/documents/method-546-determination-total-microcystins-nodularins-drinking-water-ambient-water-adda-enzyme-linked-immunosorbent-assay.pdf.

[32] Marcuello C. (2022). Present and Future Opportunities in the Use of Atomic Force Microscopy to Address the Physico-Chemical Properties of Aquatic Ecosystems at the Nanoscale Level. Int. Aquat. Res..

[33] Zhou Q., Zhang J., Fu J., Shi J., Jiang G. (2008). Biomonitoring: An Appealing Tool for Assessment of Metal Pollution in the Aquatic Ecosystem. Anal. Chim. Acta.

[34] Ceballos-Laita L., Marcuello C., Lostao A., Calvo-Begueria L., Velazquez-Campoy A., Bes M.T., Fillat M.F., Peleato M.-L. (2017). Microcystin-LR Binds Iron, and Iron Promotes Self-Assembly. Environ. Sci. Technol..

[35] Pillet F., Dague E., Ilić J.P., Ružić I., Rols M.-P., DeNardis N.I. (2019). Changes in Nanomechanical Properties and Adhesion Dynamics of Algal Cells during Their Growth. Bioelectrochem. Amst. Neth..

[36] Respondek Z., Jerz D., Świsłowski P., Rajfur M. (2022). Active Biomonitoring of Heavy Metal Concentrations in Aquatic Environment Using Mosses and Algae. Water.

[37] Jin C., Mesquita M.M.F., Deglint J.L., Emelko M.B., Wong A. (2018). Quantification of Cyanobacterial Cells via a Novel Imaging-Driven Technique with an Integrated Fluorescence Signature. Sci. Rep..

[38] Billi D., Grilli Caiola M., Paolozzi L., Ghelardini P. (1998). A Method for DNA Extraction from the Desert Cyanobacterium Chroococcidiopsis and Its Application to Identification of FtsZ. Appl. Environ. Microbiol..

[39] Fiore M.F., Moon D.H., Tsai S.M., Lee H., Trevors J.T. (2000). Miniprep DNA Isolation from Unicellular and Filamentous Cyanobacteria. J. Microbiol. Methods.

[40] Zhang J.-Y., Lin G.-M., Xing W.-Y., Zhang C.-C. (2018). Diversity of Growth Patterns Probed in Live Cyanobacterial Cells Using a Fluorescent Analog of a Peptidoglycan Precursor. Front. Microbiol..

[41] Islam M.S., Aryasomayajula A., Selvaganapathy P.R. (2017). A Review on Macroscale and Microscale Cell Lysis Methods. Micromachines.

[42] Kim I.S., Nguyen G.H., Kim S., Lee J., Yu H.-W. (2009). Evaluation of Methods for Cyanobacterial Cell Lysis and Toxin (Microcystin-LR) Extraction Using Chromatographic and Mass Spectrometric Analyses. Environ. Eng. Res..

[43] Mehta K.K., Evitt N.H., Swartz J.R. (2015). Chemical Lysis of Cyanobacteria. J. Biol. Eng..

[44] Bhardwaj B., Agrawal A., Ledwani L., Vaiphei S.T., Jain S. (2019). An Efficient Method for DNA Extraction from Cyanobacteria Isolated from Hypersaline and Marine Environments. J. Phycol..

[45] Kim Tiam S., Comte K., Dalle C., Duval C., Pancrace C., Gugger M., Marie B., Yéprémian C., Bernard C. (2019). Development of a New Extraction Method Based on High-Intensity Ultra-Sonication to Study RNA Regulation of the Filamentous Cyanobacteria Planktothrix. PLoS ONE.

[46] Singh S.P., Rastogi R.P., Häder D.-P., Sinha R.P. (2011). An Improved Method for Genomic DNA Extraction from Cyanobacteria. World J. Microbiol. Biotechnol..

[47] Xiao Z., Yang G., Yan D., Li S., Chen Z., Li W., Wu Y., Chen H. (2019). Effects of Diverse Materials-Based Methods on DNA Extraction for Clostridium Difficle from Stool Samples. Mater. Express.

[48] Emaus M.N., Anderson J.L. (2020). Simultaneous Cell Lysis and DNA Extraction from Whole Blood Using Magnetic Ionic Liquids. Anal. Bioanal. Chem..

[49] Schrader C., Schielke A., Ellerbroek L., Johne R. (2012). PCR Inhibitors—Occurrence, Properties and Removal. J. Appl. Microbiol..

[50] Koshy L., Anju A.L., Harikrishnan S., Kutty V.R., Jissa V.T., Kurikesu I., Jayachandran P., Jayakumaran Nair A., Gangaprasad A., Nair G.M. (2017). Evaluating Genomic DNA Extraction Methods from Human Whole Blood Using Endpoint and Real-Time PCR Assays. Mol. Biol. Rep..

[51] Brown R.B., Audet J. (2008). Current Techniques for Single-Cell Lysis. J. R. Soc. Interface.

[52] Pandur Ž., Dular M., Kostanjšek R., Stopar D. (2022). Bacterial Cell Wall Material Properties Determine E. Coli Resistance to Sonolysis. Ultrason. Sonochem..

[53] Wu X., Zarka A., Boussiba S. (2000). A Simplified Protocol for Preparing DNA from Filamentous Cyanobacteria. Plant Mol. Biol. Rep..

[54] Rajasekhar P., Fan L., Nguyen T., Roddick F.A. (2012). A Review of the Use of Sonication to Control Cyanobacterial Blooms. Water Res..

[55] Gaget V., Keulen A., Lau M., Monis P., Brookes J.D. (2017). DNA Extraction from Benthic Cyanobacteria: Comparative Assessment and Optimization. J. Appl. Microbiol..

[56] Greenstein K.E., Zamyadi A., Wert E.C. (2021). Comparative Assessment of Physical and Chemical Cyanobacteria Cell Lysis Methods for Total Microcystin-LR Analysis. Toxins.

[57] Danaeifar M. (2022). New Horizons in Developing Cell Lysis Methods: A Review. Biotechnol. Bioeng..

[58] Dahlgren K.K., Gates C., Lee T., Cameron J.C. (2021). Proximity-Based Proteomics Reveals the Thylakoid Lumen Proteome in the Cyanobacterium Synechococcus Sp. PCC 7002. Photosynth. Res..

[59] Shinde S., Singapuri S., Jiang Z., Long B., Wilcox D., Klatt C., Jones J.A., Yuan J.S., Wang X. (2022). Thermodynamics Contributes to High Limonene Productivity in Cyanobacteria. Metab. Eng. Commun..

[60] De Porcellinis A., Frigaard N.-U., Sakuragi Y. (2017). Determination of the Glycogen Content in Cyanobacteria. J. Vis. Exp. JoVE.

[61] Hood R.D., Higgins S.A., Flamholz A., Nichols R.J., Savage D.F. (2016). The Stringent Response Regulates Adaptation to Darkness in the Cyanobacterium Synechococcus Elongatus. Proc. Natl. Acad. Sci. USA.

[62] Bürgmann H., Pesaro M., Widmer F., Zeyer J. (2001). A Strategy for Optimizing Quality and Quantity of DNA Extracted from Soil. J. Microbiol. Methods.

[63] Scharf S., Bartels A., Kondakci M., Pfeffer K., Henrich B., Haas R. (2020). Introduction of a Bead Beating Step Improves Fungal DNA Extraction from Selected Patient Specimens. Int. J. Med. Microbiol..

[64] D’Hondt E., Martín-Juárez J., Bolado S., Kasperoviciene J., Koreiviene J., Sulcius S., Elst K., Bastiaens L., Gonzalez-Fernandez C., Muñoz R. (2017). 6—Cell Disruption Technologies. Microalgae-Based Biofuels and Bioproducts.

[65] Morin N., Vallaeys T., Hendrickx L., Natalie L., Wilmotte A. (2010). An Efficient DNA Isolation Protocol for Filamentous Cyanobacteria of the Genus Arthrospira. J. Microbiol. Methods.

[66] Wu X., Hou L., Lin X., Xie Z., Wang X., Chen X. (2019). Chapter 12—Application of Novel Nanomaterials for Chemo- and Biosensing of Algal Toxins in Shellfish and Water. Novel Nanomaterials for Biomedical, Environmental and Energy Applications.

[67] Verschoor M.J., Powe C.R., McQuay E., Schiff S.L., Venkiteswaran J.J., Li J., Molot L.A. (2017). Internal Iron Loading and Warm Temperatures Are Preconditions for Cyanobacterial Dominance in Embayments along Georgian Bay, Great Lakes. Can. J. Fish. Aquat. Sci..

[68] McKay R.M., Frenken T., Diep N., Cody W.R., Crevecoeur S., Dove A., Drouillard K.G., Ortiz X., Wintermute J., Zastepa A. (2020). Bloom Announcement: An Early Autumn Cyanobacterial Bloom Co-Dominated by Aphanizomenon Flos-Aquae and Planktothrix Agardhii in an Agriculturally-Influenced Great Lakes Tributary (Thames River, Ontario, Canada). Data Brief.

[69] Bukowska A., Bielczyńska A., Karnkowska A., Chróst R.J., Jasser I. (2014). Molecular (PCR-DGGE) versus Morphological Approach: Analysis of Taxonomic Composition of Potentially Toxic Cyanobacteria in Freshwater Lakes. Aquat. Biosyst..

[70] Almuhtaram H., Kibuye F.A., Ajjampur S., Glover C.M., Hofmann R., Gaget V., Owen C., Wert E.C., Zamyadi A. (2021). State of Knowledge on Early Warning Tools for Cyanobacteria Detection. Ecol. Indic..

[71] Chen K., Allen J., Lu J. (2017). Community Structures of Phytoplankton with Emphasis on Toxic Cyanobacteria in an Ohio Inland Lake during Bloom Season. J. Water Resour. Prot..

[72] Vadrucci M.R., Roselli L., Castelluccia D., Di Festa T., Donadei D., Florio M., Longo E., D’Arpa S., Maci F., Ranieri S. (2018). PhytoNumb3rs: An Easy-to-Use Computer Toolkit for Counting Microalgae by the Utermöhl Method. Ecol. Inform..

[73] Doktycz M.J., Sullivan C.J., Hoyt P.R., Pelletier D.A., Wu S., Allison D.P. (2003). AFM Imaging of Bacteria in Liquid Media Immobilized on Gelatin Coated Mica Surfaces. Ultramicroscopy.

[74] Guan D., Barraud C., Charlaix E., Tong P. (2017). Noncontact Viscoelastic Measurement of Polymer Thin Films in a Liquid Medium Using Long-Needle Atomic Force Microscopy. Langmuir.

[75] Qian S.S., Chaffin J.D., DuFour M.R., Sherman J.J., Golnick P.C., Collier C.D., Nummer S.A., Margida M.G. (2015). Quantifying and Reducing Uncertainty in Estimated Microcystin Concentrations from the ELISA Method. Environ. Sci. Technol..

[76] Fischer W.J., Garthwaite I., Miles C.O., Ross K.M., Aggen J.B., Chamberlin A.R., Towers N.R., Dietrich D.R. (2001). Congener-Independent Immunoassay for Microcystins and Nodularins. Environ. Sci. Technol..

[77] Lu J., Struewing I., Wymer L., Tettenhorst D.R., Shoemaker J., Allen J. (2020). Use of QPCR and RT-QPCR for Monitoring Variations of Microcystin Producers and as an Early Warning System to Predict Toxin Production in an Ohio Inland Lake. Water Res..

[78] Health Canada Guidelines for Canadian Drinking Water Quality: Guideline Technical Document—Cyanobacterial Toxins. https://www.canada.ca/en/health-canada/services/publications/healthy-living/guidelines-canadian-drinking-water-quality-guideline-technical-document-cyanobacterial-toxins-document.html.

[79] Douglas Greene S.B., LeFevre G.H., Markfort C.D. (2021). Improving the Spatial and Temporal Monitoring of Cyanotoxins in Iowa Lakes Using a Multiscale and Multi-Modal Monitoring Approach. Sci. Total Environ..

[80] Mountfort D.O., Holland P., Sprosen J. (2005). Method for Detecting Classes of Microcystins by Combination of Protein Phosphatase Inhibition Assay and ELISA: Comparison with LC-MS. Toxicon.

[81] He X., Stanford B.D., Adams C., Rosenfeldt E.J., Wert E.C. (2017). Varied Influence of Microcystin Structural Difference on ELISA Cross-Reactivity and Chlorination Efficiency of Congener Mixtures. Water Res..

[82] Aranda-Rodriguez R., Jin Z. (2011). Evaluation of Field Test Kits to Detect Microcystins: 2010 Study.

[83] Birbeck J.A., Westrick J.A., O’Neill G.M., Spies B., Szlag D.C. (2019). Comparative Analysis of Microcystin Prevalence in Michigan Lakes by Online Concentration LC/MS/MS and ELISA. Toxins.

[84] Coskun O. (2016). Separation Techniques: Chromatography. North. Clin. Istanb..

[85] Flores C., Caixach J. (2015). An Integrated Strategy for Rapid and Accurate Determination of Free and Cell-Bound Microcystins and Related Peptides in Natural Blooms by Liquid Chromatography–Electrospray-High Resolution Mass Spectrometry and Matrix-Assisted Laser Desorption/Ionization Time-of-Flight/Time-of-Flight Mass Spectrometry Using Both Positive and Negative Ionization Modes. J. Chromatogr. A.

[86] Filatova D., Núñez O., Farré M. (2020). Ultra-Trace Analysis of Cyanotoxins by Liquid Chromatography Coupled to High-Resolution Mass Spectrometry. Toxins.

[87] Greer B., McNamee S.E., Boots B., Cimarelli L., Guillebault D., Helmi K., Marcheggiani S., Panaiotov S., Breitenbach U., Akçaalan R. (2016). A Validated UPLC-MS/MS Method for the Surveillance of Ten Aquatic Biotoxins in European Brackish and Freshwater Systems. Harmful Algae.

[88] Lawrence J.F., Niedzwiadek B., Menard C., Lau B.P.Y., Lewis D., Kuper-Goodman T., Carbone S., Holmes C. (2001). Comparison of Liquid Chromatography/Mass Spectrometry, ELISA, and Phosphatase Assay for the Determination of Microcystins in Blue-Green Algae Products. J. AOAC Int..

[89] Kumar P., Rautela A., Kesari V., Szlag D., Westrick J., Kumar S. (2020). Recent Developments in the Methods of Quantitative Analysis of Microcystins. J. Biochem. Mol. Toxicol..

[90] Gaget V., Lau M., Sendall B., Froscio S., Humpage A.R. (2017). Cyanotoxins: Which Detection Technique for an Optimum Risk Assessment?. Water Res..

[91] Kralik P., Ricchi M. (2017). A Basic Guide to Real Time PCR in Microbial Diagnostics: Definitions, Parameters, and Everything. Front. Microbiol..

[92] Pinto F., Pacheco C.C., Ferreira D., Moradas-Ferreira P., Tamagnini P. (2012). Selection of Suitable Reference Genes for RT-QPCR Analyses in Cyanobacteria. PLoS ONE.

[93] Barón-Sola Á., Campo F.F.d., Sanz-Alférez S. (2016). Dynamics of Cylindrospermopsin Production and Toxin Gene Expression in Aphanizomenon Ovalisporum. Adv. Microbiol..

[94] Chiu Y.-T., Chen Y.-H., Wang T.-S., Yen H.-K., Lin T.-F. (2017). A QPCR-Based Tool to Diagnose the Presence of Harmful Cyanobacteria and Cyanotoxins in Drinking Water Sources. Int. J. Environ. Res. Public. Health.

[95] Oh K.-H., Jeong D.-H., Shin S.-H., Cho Y.-C. (2012). Simultaneous Quantification of Cyanobacteria and Microcystis Spp. Using Real-Time PCR. J. Microbiol. Biotechnol..

[96] Te S.H., Chen E.Y., Gin K.Y.-H. (2015). Comparison of Quantitative PCR and Droplet Digital PCR Multiplex Assays for Two Genera of Bloom-Forming Cyanobacteria, Cylindrospermopsis and Microcystis. Appl. Environ. Microbiol..

[97] Luo X., Li J., Chang T., He H., Zhao Y., Yang X., Zhao Y., Xu Y. (2019). Stable Reference Gene Selection for RT-QPCR Analysis in *Synechococcus Elongatus* PCC 7942 under Abiotic Stresses. BioMed Res. Int..

[98] Rinta-Kanto J.M., Ouellette A.J.A., Boyer G.L., Twiss M.R., Bridgeman T.B., Wilhelm S.W. (2005). Quantification of Toxic Microcystis Spp. during the 2003 and 2004 Blooms in Western Lake Erie Using Quantitative Real-Time PCR. Environ. Sci. Technol..

[99] Khomutovska N., Sandzewicz M., Łach Ł., Suska-Malawska M., Chmielewska M., Mazur-Marzec H., Cegłowska M., Niyatbekov T., Wood S.A., Puddick J. (2020). Limited Microcystin, Anatoxin and Cylindrospermopsin Production by Cyanobacteria from Microbial Mats in Cold Deserts. Toxins.

[100] Zupančič M., Kogovšek P., Šter T., Rekar Š.R., Cerasino L., Baebler Š., Klemenčič A.K., Eleršek T. (2021). Potentially Toxic Planktic and Benthic Cyanobacteria in Slovenian Freshwater Bodies: Detection by Quantitative PCR. Toxins.

[101] Li X., Huo S., Zhang J., Ma C., Xiao Z., Zhang H., Xi B., Xia X. (2019). Metabarcoding Reveals a More Complex Cyanobacterial Community than Morphological Identification. Ecol. Indic..

[102] Casero M.C., Velázquez D., Medina-Cobo M., Quesada A., Cirés S. (2019). Unmasking the Identity of Toxigenic Cyanobacteria Driving a Multi-Toxin Bloom by High-Throughput Sequencing of Cyanotoxins Genes and 16S RRNA Metabarcoding. Sci. Total Environ..

[103] MacKeigan P.W., Garner R.E., Monchamp M.-È., Walsh D.A., Onana V.E., Kraemer S.A., Pick F.R., Beisner B.E., Agbeti M.D., da Costa N.B. (2022). Comparing Microscopy and DNA Metabarcoding Techniques for Identifying Cyanobacteria Assemblages across Hundreds of Lakes. Harmful Algae.

[104] Jungblut A.-D., Neilan B.A. (2006). Molecular Identification and Evolution of the Cyclic Peptide Hepatotoxins, Microcystin and Nodularin, Synthetase Genes in Three Orders of Cyanobacteria. Arch. Microbiol..

[105] Tillett D., Parker D.L., Neilan B.A. (2001). Detection of Toxigenicity by a Probe for the Microcystin Synthetase A Gene (McyA) of the Cyanobacterial Genus Microcystis: Comparison of Toxicities with 16S RRNA and Phycocyanin Operon (Phycocyanin Intergenic Spacer) Phylogenies. Appl. Environ. Microbiol..

[106] Furukawa K., Noda N., Tsuneda S., Saito T., Itayama T., Inamori Y. (2006). Highly Sensitive Real-Time PCR Assay for Quantification of Toxic Cyanobacteria Based on Microcystin Synthetase A Gene. J. Biosci. Bioeng..

[107] Ngwa F., Madramootoo C., Jabaji S. (2014). Monitoring Toxigenic Microcystis Strains in the Missisquoi Bay, Quebec, by PCR Targeting Multiple Toxic Gene Loci. Environ. Toxicol..

[108] Ngwa F.F., Madramootoo C.A., Jabaji S. (2014). Development and Application of a Multiplex QPCR Technique to Detect Multiple Microcystin-Producing Cyanobacterial Genera in a Canadian Freshwater Lake. J. Appl. Phycol..

[109] Gupta R.S. (2018). Impact of Genomics on Clarifying the Evolutionary Relationships amongst Mycobacteria: Identification of Molecular Signatures Specific for the Tuberculosis-Complex of Bacteria with Potential Applications for Novel Diagnostics and Therapeutics. High-Throughput.

[110] Gupta R.S., Mathews D.W. (2010). Signature Proteins for the Major Clades of Cyanobacteria. BMC Evol. Biol..

[111] Rinta-Kanto J.M., Konopko E.A., DeBruyn J.M., Bourbonniere R.A., Boyer G.L., Wilhelm S.W. (2009). Lake Erie Microcystis: Relationship between Microcystin Production, Dynamics of Genotypes and Environmental Parameters in a Large Lake. Harmful Algae.

[112] Duan X., Zhang C., Struewing I., Li X., Allen J., Lu J. (2022). Cyanotoxin-Encoding Genes as Powerful Predictors of Cyanotoxin Production during Harmful Cyanobacterial Blooms in an Inland Freshwater Lake: Evaluating a Novel Early-Warning System. Sci. Total Environ..

[113] Crevecoeur S., Edge T.A., Watson L.C., Watson S.B., Greer C.W., Ciborowski J.J.H., Diep N., Dove A., Drouillard K.G., Frenken T. (2023). Spatio-Temporal Connectivity of the Aquatic Microbiome Associated with Cyanobacterial Blooms along a Great Lake Riverine-Lacustrine Continuum. Front. Microbiol..

[114] Eiler A., Gonzalez-Rey C., Allen S., Bertilsson S. (2007). Growth Response of Vibrio Cholerae and Other Vibrio Spp. to Cyanobacterial Dissolved Organic Matter and Temperature in Brackish Water. FEMS Microbiol. Ecol..

[115] Gaget V., Hobson P., Keulen A., Newton K., Monis P., Humpage A.R., Weyrich L.S., Brookes J.D. (2020). Toolbox for the Sampling and Monitoring of Benthic Cyanobacteria. Water Res..

[116] Dorevitch S., Shrestha A., DeFlorio-Barker S., Breitenbach C., Heimler I. (2017). Monitoring Urban Beaches with QPCR vs. Culture Measures of Fecal Indicator Bacteria: Implications for Public Notification. Environ. Health.

[117] Saleem F., Edge T.A., Schellhorn H.E. (2022). Validation of QPCR Method for Enterococci Quantification at Toronto Beaches: Application for Rapid Recreational Water Monitoring. J. Gt. Lakes Res..

[118] Knob R., Hanson R.L., Tateoka O.B., Wood R.L., Guerrero-Arguero I., Robison R.A., Pitt W.G., Woolley A.T. (2018). Sequence-Specific Sepsis-Related DNA Capture and Fluorescent Labeling in Monoliths Prepared by Single-Step Photopolymerization in Microfluidic Devices. J. Chromatogr. A.

[119] Wei Y.-J., Zhao Y.-N., Zhang X., Wei X., Chen M.-L., Chen X.-W. (2023). Biochemical Analysis Based on Optical Detection Integrated Microfluidic Chip. TrAC Trends Anal. Chem..

[120] Jiao H. (2013). Handheld Microfluidic Device for Cyanobacteria Toxin Detection and Monitoring|Phase I.

[121] Jiao H. (2017). Handheld Microfluidic Device for Cyanobacteria Toxin Detection and Monitoring | Phase II.

[122] Blanco Y., Quesada A., Gallardo-Carreño I., Aguirre J., Parro V. (2015). CYANOCHIP: An Antibody Microarray for High-Taxonomical-Resolution Cyanobacterial Monitoring. Environ. Sci. Technol..

[123] Blanco Y., Moreno-Paz M., Parro V. (2017). Experimental Protocol for Detecting Cyanobacteria in Liquid and Solid Samples with an Antibody Microarray Chip. J. Vis. Exp. JoVE.

[124] Ölcer Z., Esen E., Ersoy A., Budak S., Sever Kaya D., Yağmur Gök M., Barut S., Üstek D., Uludag Y. (2015). Microfluidics and Nanoparticles Based Amperometric Biosensor for the Detection of Cyanobacteria (Planktothrix Agardhii NIVA-CYA 116) DNA. Biosens. Bioelectron..

[125] Johnson B.N., Mutharasan R. (2013). A Cantilever Biosensor-Based Assay for Toxin-Producing Cyanobacteria Microcystis Aeruginosa Using 16S RRNA. Environ. Sci. Technol..

[126] Rantala A., Rizzi E., Castiglioni B., De Bellis G., Sivonen K. (2008). Identification of Hepatotoxin-Producing Cyanobacteria by DNA-Chip. Environ. Microbiol..

[127] Sipari H., Rantala-Ylinen A., Jokela J., Oksanen I., Sivonen K. (2010). Development of a Chip Assay and Quantitative PCR for Detecting Microcystin Synthetase E Gene Expression. Appl. Environ. Microbiol..

[128] Khan M., Mao S., Li W., Lin J.-M. (2018). Microfluidic Devices in the Fast-Growing Domain of Single-Cell Analysis. Chem.—Eur. J..

[129] Shi K., Zhang Y., Qin B., Zhou B. (2019). Remote Sensing of Cyanobacterial Blooms in Inland Waters: Present Knowledge and Future Challenges. Sci. Bull..

[130] Mishra S., Stumpf R.P., Schaeffer B.A., Werdell P.J., Loftin K.A., Meredith A. (2019). Measurement of Cyanobacterial Bloom Magnitude Using Satellite Remote Sensing. Sci. Rep..

[131] Mishra S., Stumpf R.P., Schaeffer B., Werdell P.J., Loftin K.A., Meredith A. (2021). Evaluation of a Satellite-Based Cyanobacteria Bloom Detection Algorithm Using Field-Measured Microcystin Data. Sci. Total Environ..

[132] Coffer M.M., Schaeffer B.A., Darling J.A., Urquhart E.A., Salls W.B. (2020). Quantifying National and Regional Cyanobacterial Occurrence in US Lakes Using Satellite Remote Sensing. Ecol. Indic..

[133] Stumpf R.P., Davis T.W., Wynne T.T., Graham J.L., Loftin K.A., Johengen T.H., Gossiaux D., Palladino D., Burtner A. (2016). Challenges for Mapping Cyanotoxin Patterns from Remote Sensing of Cyanobacteria. Harmful Algae.

[134] Beck R., Xu M., Zhan S., Liu H., Johansen R.A., Tong S., Yang B., Shu S., Wu Q., Wang S. (2017). Comparison of Satellite Reflectance Algorithms for Estimating Phycocyanin Values and Cyanobacterial Total Biovolume in a Temperate Reservoir Using Coincident Hyperspectral Aircraft Imagery and Dense Coincident Surface Observations. Remote Sens..

[135] United States Environmental Protection Agency Cyanobacteria Assessment Network (CyAN). https://www.epa.gov/water-research/cyanobacteria-assessment-network-cyan.

[136] Environment and Climate Change Canada EOLakeWatch: Satellite Earth Observations for Lake Monitoring. https://www.canada.ca/en/environment-climate-change/services/water-overview/satellite-earth-observations-lake-monitoring.html.

[137] Environment and Climate Change Canada Remote Sensing of Algal Blooms. https://www.canada.ca/en/environment-climate-change/services/water-overview/satellite-earth-observations-lake-monitoring/remote-sensing-algal-blooms.html.

[138] Binding C.E., Zastepa A., Zeng C. (2019). The Impact of Phytoplankton Community Composition on Optical Properties and Satellite Observations of the 2017 Western Lake Erie Algal Bloom. J. Gt. Lakes Res..

[139] Cao H., Recknagel F., Bartkow M. (2016). Spatially-Explicit Forecasting of Cyanobacteria Assemblages in Freshwater Lakes by Multi-Objective Hybrid Evolutionary Algorithms. Ecol. Model..

[140] Chen Q., Guan T., Yun L., Li R., Recknagel F. (2015). Online Forecasting Chlorophyll a Concentrations by an Auto-Regressive Integrated Moving Average Model: Feasibilities and Potentials. Harmful Algae.

[141] Baek S.-S., Pyo J., Pachepsky Y., Park Y., Ligaray M., Ahn C.-Y., Kim Y.-H., Ahn Chun J., Hwa Cho K. (2020). Identification and Enumeration of Cyanobacteria Species Using a Deep Neural Network. Ecol. Indic..

[142] Gaur A., Pant G., Jalal A.S. (2022). Computer-Aided Cyanobacterial Harmful Algae Blooms (CyanoHABs) Studies Based on Fused Artificial Intelligence (AI) Models. Algal Res..

[143] Zhu M., Wang J., Yang X., Zhang Y., Zhang L., Ren H., Wu B., Ye L. (2022). A Review of the Application of Machine Learning in Water Quality Evaluation. Eco-Environ. Health.

[144] Xu T., Coco G., Neale M. (2020). A Predictive Model of Recreational Water Quality Based on Adaptive Synthetic Sampling Algorithms and Machine Learning. Water Res..

[145] Lowe M., Qin R., Mao X. (2022). A Review on Machine Learning, Artificial Intelligence, and Smart Technology in Water Treatment and Monitoring. Water.

